# Carotenoids—Antioxidant Properties

**DOI:** 10.3390/antiox7020028

**Published:** 2018-02-11

**Authors:** Andrew J. Young, Gordon L. Lowe

**Affiliations:** 1 School of Natural Sciences and Psychology, Liverpool John Moores University, Byrom Street, Liverpool L3 3AF, UK; 2 School of Pharmacy and Biomolecular Sciences, Liverpool John Moores University, Byrom Street, Liverpool L3 3AF, UK

The carotenoid group of pigments are ubiquitous in nature and more than 600 different carotenoids have been identified and characterized [1]. They are responsible for pigmentation in animals, plants, and microorganisms, but crucially also serve important, often critical, roles in biological systems. Indeed, in recent years most attention focused on this group of pigments has concerned understanding their function, especially as antioxidants. The “core” structural element of carotenoids is a polyene backbone consisting of a series of conjugated C=C bonds. This particular feature is primarily responsible for both their pigmenting properties and the ability of many of these compounds to interact with free radicals and singlet oxygen and therefore act as effective antioxidants. Modifications to this polyene backbone, altering the number of conjugated double bonds together with the addition of oxygen functional groups, in turn, alter the reactivity of carotenoids. Importantly, the function of carotenoids is also substantially affected by their immediate environment, which, in turn, is dependent on their structure (e.g., [2]). This is arguably most evident in photosynthetic systems in higher plants and algae where xanthophylls are restricted to light-harvesting complexes (performing both light-capture and photoprotective roles), whilst β-carotene is found in reaction centers (a protective role) (e.g., [3,4]).

Whilst carotenoids are widely distributed across natural systems, research has largely concentrated on just a few compounds that are involved in aspects of human health (notably the dietary compounds β-carotene, lutein, and lycopene) or in photosynthetic processes in plants and photosynthetic bacteria (e.g., β-carotene, spheroidene, lutein, violaxanthin, and zeaxanthin). In human health, large-scale epidemiological studies have demonstrated a strong association between diets rich in fruit and vegetables (including the “Mediterranean” diet) and reductions in certain diseases, including some cancers and heart disease [5]. This in turn led to large dietary intervention studies, some of which explored the use of high doses of β-carotene in smokers and asbestos workers. Two of the most influential studies were the Beta-Carotene and Retinol Efficacy Trial (CARET [6]) and the Alpha-Tocopherol Beta-Carotene Cancer Prevention Trial (ATBC [7]). However, the results from such studies appeared to contradict the dietary studies that preceded them, highlighting the need to better understand how carotenoids behave in biological, especially human, systems and, indeed, whether carotenoids can act as both antioxidants and pro-oxidants under different conditions.

This special issue consists of a set of articles which highlight some of these recent advances concerning the antioxidant properties of carotenoids, reflecting the wide range of studies on this fascinating group of natural products. Edge and Truscott [8] review the most recent work on the interaction between singlet oxygen, free radicals, and carotenoids and retinoids. Whilst the antioxidant properties of these compounds are well-known, the article highlights some important, often less well-studied, issues. Recent research by the authors demonstrates that carotenoids can switch from antioxidant to pro-oxidant behavior as a function of oxygen concentration. Employing a cell-based model system, they observed total protection from exposure to high energy γ-radiation by lycopene at 0% oxygen, but zero protection at 100% oxygen. This may have implications for the behavour of carotenoids in tissues where different partial pressures of oxygen are present. The physical “organization” (e.g., the tendency for carotenoids to aggregate in different solvents) of the carotenoid is an important consideration that affects its antioxidant abilities, through its interactions with reactive oxygen species themselves as well as with other antioxidants such as α-tocopherol and vitamin C. The antioxidant properties of the carotenoid astaxanthin are studied by Focsan et al. [9]. This pigment is bound to the white muscle of salmonids, imparting the characteristic pink coloration of the fish, and is found in the pigment-protein complexes of the carapace of a number of crustacea. Astaxanthin also accumulates in the freshwater microalga *Haematococcus pluvialis* under stress conditions (e.g., nutrient deprivation, exposure to high irradiances, or in the presence of reactive oxygen species). Using a range of techniques including electron paramagnetic resonance, Foscan and colleagues [9] indicate that a range of factors influence the antioxidant activity of astaxanthin. These include: the formation of chelate complexes with metals; esterification and its inability to aggregate in the ester form; a high oxidation potential; and the formation of neutral radicals under high irradiation in the presence of metal ions. 

As these papers illustrate, there is no doubt that the interaction of carotenoids with reactive oxidizing species is highly complex. The fate of these carotenoids and the properties of the resulting reaction products, including geometric isomers, adducts, and breakdown or cleavage compounds are still relatively poorly understood. In this special issue, two papers consider separate aspects of this. First, Haider and colleagues [10] explore the potential genotoxic and cytotoxic roles of oxidative breakdown products of carotenoids. The pro-oxidant effects resulting from exposure to high doses of carotenoids seen in vivo (as in the CARET and ATBC trials [6,7]), or enhanced DNA damage seen in in vitro studies (e.g., [11]) are often associated with the accumulation and subsequent deleterious actions of a range of putative breakdown products. Haider et al. [10] found that low doses (1 µM) of cleavage products of β-carotene (generated by hypochlorite treatment) induced significant levels of DNA strand breaks in primary pneumocyte type II cells that were subjected to oxidative stress. By contrast, β-carotene itself acted as an effective antioxidant and cytotoxic effects were only seen at much higher concentrations (50 μM). The in vivo oxidative generation of geometric isomers of another major dietary carotenoid, lycopene, is considered by Graham et al. [12]. In vitro studies have demonstrated that exposure to the complex mixture of free radicals found in cigarette smoke induces the bleaching of carotenoids, such as lycopene and β-carotene, via a series of reactions including cleavage and isomerization [13,14]. The detection of such reaction products in vivo is especially challenging due to their (often) transient nature and trace levels. Graham et al. [12] found that the plasma of smokers contained elevated proportions of (13Z)-lycopene relative to the other geometric isomers of this carotenoid. This finding is consistent with in vitro observations that this particular, energetically-unfavored, geometric form was preferentially generated in the presence of cigarette smoke [13]. Further work is needed to determine the full range of reaction products of dietary carotenoids when exposed to reactive oxygen species, elucidate the pathways by which such degradation occurs, and better understand their possible function. 

The role of carotenoids in the human macula is discussed by Gong et al. [15]. The xanthophylls lutein and zeaxanthin are accumulated within and protect the macula. This study examined the behavour of three dietary carotenoids, namely, β-carotene, lycopene, and lutein, in retinal pigment epithelial cells. Lutein and lycopene, but not β-carotene, inhibited the growth of undifferentiated ARPE-19 cells. Moreover, cell viability was decreased under hypoxic conditions. It is worthwhile noting that the macula carotenoids (lutein and zeaxanthin) also have well-defined functional roles in higher plant photosynthesis, in both light-capture and energy-quenching [3]. The ability of these molecules to function in plants and humans alike is dependent upon the same chemical and physical properties.

Carotenoids are widely distributed across the natural world and to reflect this, Galasso et al. [16] review the occurrence and diversity of carotenoids in the marine environment as well as their potential for economic exploitation (e.g., as natural sources of pigments for food and feed industries or as a source of antioxidants). Carotenoids are recognized as the most common class of pigments in the marine environment, with a much greater diversity of structures than that seen in the terrestrial environment [1]. However, beyond a handful of compounds such as astaxanthin and fucoxanthin, they remain relatively poorly studied. Continuing the economic theme, Fu et al. [17] examine the distribution of pigments and their antioxidant activities in durum wheat milling fractions.

In conclusion, carotenoids remain a fascinating group of natural pigments. Not only are they responsible for a broad array of coloration in nature, but, more importantly, they have key functional roles in biology. Studies on their function in human health and disease have all too often focused solely on what might be regarding as a hunt for a “magic bullet” effect, i.e., a particular carotenoid (e.g., β-carotene) is found in “healthy” diets and, as it is an antioxidant (at least in vitro), it is assumed that high doses must have a beneficial effect. Sadly, all too often this approach has been shown to be far too simplistic, neglecting as it does the interaction with other dietary components (including other antioxidants) and the fate of the antioxidants themselves, especially when present at high doses. Whilst some researchers (e.g., Truscott and Edge) have always considered some of these aspects, we are now seeing many more studies tackling these complex and technically challenging issues.
